# Decoding CAR T cell phenotype using combinatorial signaling motif libraries and machine learning

**DOI:** 10.1126/science.abq0225

**Published:** 2022-12-08

**Authors:** Kyle G. Daniels, Shangying Wang, Milos S. Simic, Hersh K. Bhargava, Sara Capponi, Yurie Tonai, Wei Yu, Simone Bianco, Wendell A. Lim

**Affiliations:** 1 Cell Design Institute and Department of Cellular and Molecular Pharmacology, University of California, San Francisco, San Francisco, CA 94158.; 2 Department of Functional Genomics and Cellular Engineering, IBM Almaden Research Center, San Jose, CA 95120; 3 Center for Cellular Construction, San Francisco, CA, 94158

**Keywords:** Synthetic Biology, Cell Signaling, Signaling Motifs, Cancer Immunotherapy, Machine Learning

## Abstract

Chimeric antigen receptor (CAR) costimulatory domains derived from native immune receptors steer the phenotypic output of therapeutic T cells. We constructed a library of CARs containing ~2,300 synthetic costimulatory domains, built from combinations of 13 signaling motifs. These CARs promoted diverse cell fates, which were sensitive to motif combinations and configurations. Neural networks trained to decode the combinatorial grammar of CAR signaling motifs allowed extraction of key design rules. For example, non-native combinations of motifs which bind tumor necrosis factor receptor-associated factors (TRAFs) and phospholipase C gamma 1 (PLCγ1) enhanced cytotoxicity and stemness associated with effective tumor killing. Thus, libraries built from minimal building blocks of signaling, combined with machine learning, can efficiently guide engineering of receptors with desired phenotypes.

## INTRODUCTION

Chimeric antigen receptors (CARs) have demonstrated the power of synthetic signaling receptors as tools to reprogram immune cells to execute therapeutic functions, such as targeted killing of tumor cells (1). The antitumor efficacy of CARs is strongly modulated by the signaling domains that they contain. Current clinically approved CARs contain a core T cell receptor (TCR) signaling domain from CD3ζ (containing immunoreceptor tyrosine-based activation motifs (ITAMs) that recruit the kinase ZAP70) (2–4), along with a costimulatory signaling domain from either the CD28 (5, 6) or 4-1BB (7) costimulatory immune receptors (8–10). The costimulatory domains are themselves composed of multiple signaling motifs, short peptides that bind to specific downstream signaling proteins, often through modular protein interaction domains (e.g. Src Homology 2 (SH2), Src Homology 3 (SH3), or other domains (11, 12)). Such peptide signaling motifs (referred to as linear motifs) are the fundamental building blocks controlling the output of most signaling receptors. The constellation of signaling proteins recruited by a particular array of signaling motifs upon receptor stimulation is thought to shape the distinct cellular response. For example, in CARs, the 4-1BB costimulatory domain which contains binding motifs for tumor necrosis factor receptor-associated factors (TRAF) signaling adaptor proteins leads to increased T cell memory and persistence; the CD28 costimulatory domain, which contains binding motifs for phosphatidylinositol-3-kinase (PI3K), growth factor receptor-bound protein 2 (Grb2), and lymphocyte-specific protein tyrosine kinase (Lck), is associated with more effective T cell killing, but reduced long-term T cell persistence (13). Thus, signaling motifs can be thought of as the “words” that are used to compose the phenotypic “sentences” communicated through signaling domains.

A major and still mostly outstanding goal in synthetic biology is to predictably generate new cell phenotypes by altering receptor composition. For example, in cancer immunotherapy, a general goal is to enhance T cell anti-tumor cytotoxicity but to also maintain a stem-like state associated with longer-term T cell persistence. Such a phenotype is associated with effective and durable tumor clearance (higher stemness is correlated with more resistance to T cell exhaustion). Libraries of costimulatory domains have been screened for improved phenotypes (14–16). However, the costimulatory domains used were from natural immune receptors (i.e. alternative pre-existing “sentences”, to use the analogy to language). We propose that a more effective way to scan phenotypic space for synthetic receptors is to create libraries that sample new combinations of signaling motifs. Such an approach could, in principle, yield phenotypes that extend beyond those that can be generated by native receptor domains alone. Moreover, exploration of a broader range of receptor “motif space” could lead to a more systematic understanding of how different parameters of output are encoded by motif identity, combination, and order.

**Figure 1.**
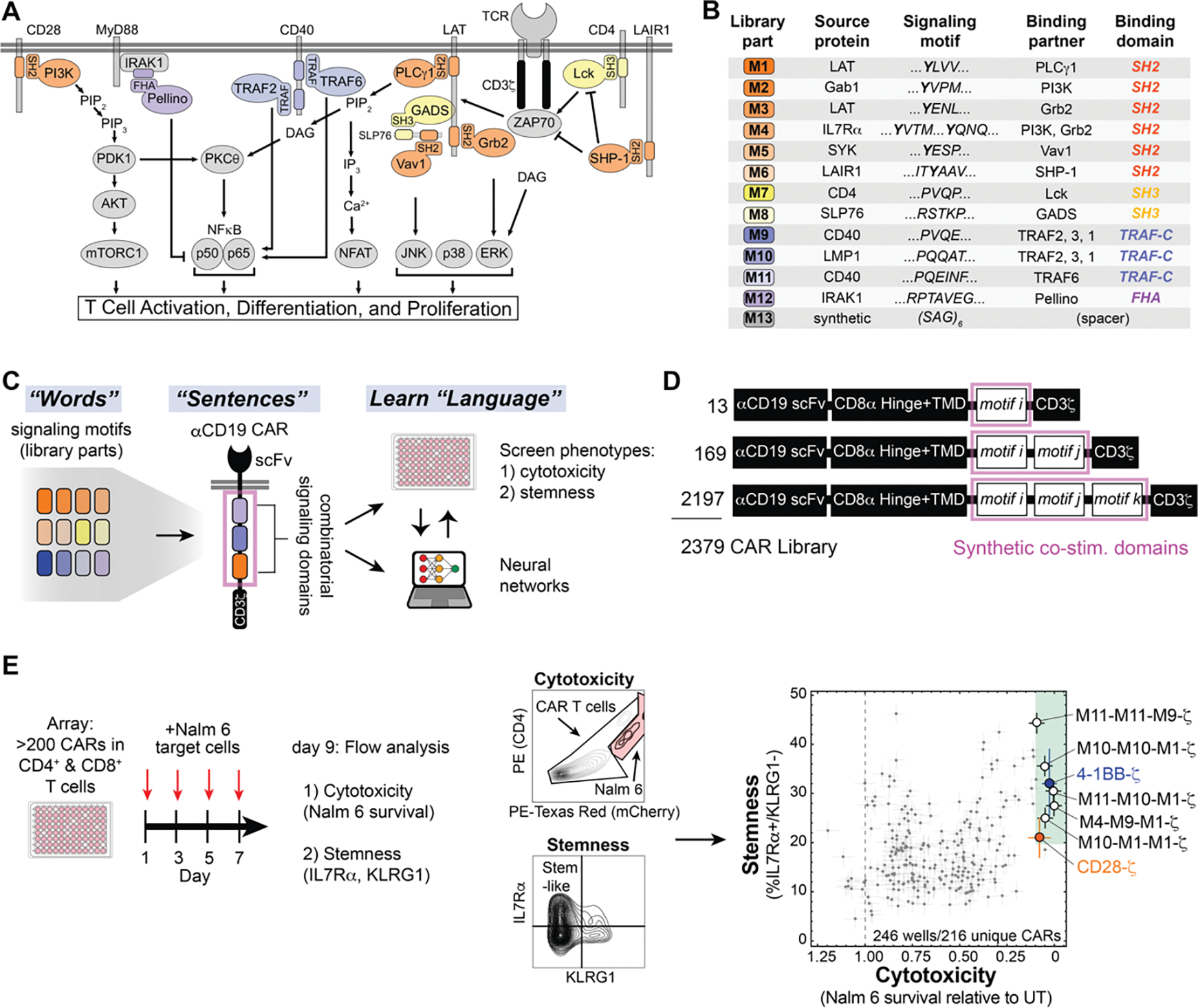
CAR costimulatory domains with synthetic signaling motif combinations generate diverse cell fates with decoupled cytotoxicity and stemness. **A**, A diverse set of proteins that function in T cell signaling are recruited by signaling motifs in the library parts. **B**, Description of library parts used in combinatorial library. Each part is 16 to 18 amino acids including the signaling motif(s) and flanking sequence. Phospho-tyrosines are shown in bold. **C**, New combinations of signaling motifs create distinct CAR signaling programs that control T cell phenotype. **D**, Schematics of αCD19 CAR with variable signaling domains. **E**, CAR T cells with various signaling motif combinations produce a broad range of cytotoxicity and stemness. CD4+ and CD8+ CAR T cells were pulsed 4 times with Nalm 6 leukemia cells and assayed for CAR T cell cytotoxicity and stemness. Errors for Nalm 6 survival, and stem-like IL7Rα+/KLRG1− population in **E** were estimated by calculating the average standard deviation for 7 CAR constructs with internal replicates in the array.

We recombined 13 signaling motifs (words) to create a CAR costimulatory domain library with randomized motif combinations (new sentences) (Fig. 1). This library of new signaling “sentences” produced a range of phenotypes, including combinations of phenotypes not observed with native signaling domains. We used neural networks to decode the language of signaling motifs, create predictive models, and extract design rules that inform the engineering of CAR signaling domains that increase cytotoxicity and stemness.

## RESULTS

### A CAR library with synthetic combinations of signaling motifs generates diverse CAR T cell cytotoxicity and memory potential

To construct a combinatorial library of CAR signaling domains, we searched the Eukaryotic Linear Motif Database (ELM) (17) and primary literature to curate a collection of 12 peptide motifs from natural signaling proteins that recruit key downstream signaling proteins that function in T cell activation. The motifs in the library recruit proteins such as phospholipase C gamma 1 (PLCγ1), TNF receptor-associated factor associated factors (TRAFs), growth factor receptor-bound protein 2 (Grb2), Grb2-related adaptor downstream of Shc (GADS), Src homology region 2 domain-containing phosphatase (SHP-1), vav guanine nucleotide exchange factor 1 (Vav1), phosphatidylinositol-3-kinase (PI3K), lymphocyte-specific protein tyrosine kinase (Lck), and Pellino protein. For example library motif 1 is derived from the linker for activation of T cells (LAT) and contains the core motif YLVV—which when tyrosine-phosphorylated, binds the N-terminal SH2 domain of PLCγ1 with high specificity(18). Motif 6, contains the motif ITYAAV from the leukocyte associated immunoglobulin-like receptor 1 (LAIR1), which binds the phosphatase SHP-1 through its SH2 domain (19). In addition to the 12 signaling motifs, we included a spacer motif as the 13^th^ component in the library. The combinatorial library was constructed within the context of an anti-CD19 CAR (containing an anti-CD19 extracellular single-chain variable fragment and a CD3ζ signaling domain). The synthetic costimulatory domains had either one, two, or three signaling motifs. The 13 motifs were randomly inserted in positions i, j, and k (Fig. 1) to yield 2379 different motif combinations (Fig. 1, B through E). To confirm that the library displayed sufficient phenotypic diversity, we first performed low resolution pooled screens, in which we transduced a mixed population of CD4+ and CD8+ primary human T cells at low multiplicity of infection and activated the pool with Nalm 6 leukemia cells (CD19+) for 8–9 days. We used FACS-based sequencing enrichment assays to observe a diverse range of phenotypic outputs for T cell proliferation, formation of central memory T cells expressing receptor-type tyrosine-protein phosphatase C (CD45RA) and lacking L-selectin (CD62L), and T cell degranulation (lysosome-associated membrane glycoprotein 1 (CD107A+) T cells, a proxy for cytotoxic response) (Fig. S1). All T cells were activated with beads displaying CD3 and CD28 to allow for viral transduction, and subsequent activation through CARs with unique signaling domains led to divergent phenotypes.

To screen the library at higher resolution, we transformed bacteria with library plasmid stocks and randomly picked colonies to select a subset of over 200 CARs from the combinatorial library to characterize in an arrayed screen (Fig. 1E). An arrayed screen—in which each CAR is studied independently— was important because immune paracrine signaling could confound analysis of pooled CAR T cell screens. We activated the CD4+ and CD8+ CAR T cells in the arrayed screen by culturing with Nalm 6 (CD19+) cells for 8 to 9 days. Four pulses of Nalm 6 cells were used to mimic longer term stimulation that can exacerbate T cell exhaustion. At the end of the co-culture, we used flow cytometry to assess cytotoxicity of the mixed CD4^+^ and CD8^+^ CAR T cell populations (based on Nalm 6 cell survival), stemness (Interleukin-7 receptor subunit alpha (IL7Rα^+^) and killer cell lectin receptor G1 (KLRG1^−^)) (20–23), and maintenance of T cell populations with markers of central memory or naïve state (CD45RA and CD62L).

The CARs in the arrayed screen displayed a range of cytotoxicity and stemness. The total naïve and central memory population was positively correlated with cytotoxicity (Fig. S2B). Stemness and the naïve population were roughly proportional across the library (Fig. S2, C and D). However, cytotoxicity and stemness were uncoupled. This observation underscores the ability of unusual combinations of motifs in costimulatory domains to drive CAR T cells to varied cell fates with particular combinations of phenotypes. Several costimulatory domains produced cytotoxicity and stemness comparable to that of 4-1BB. Many of these contained motifs that recruit both TRAFs (motif 9, motif 10, motif 11) and PLCγ1 (motif 1). For example, M10-M10-M1-ζ, M10-M1-M1-ζ, M11-M10-M1-ζ, and M4-M9-M1-ζ all promoted cytotoxicity and stemness.

### Neural networks predict the CAR T cell cytotoxicity and memory potential encoded by combinations of signaling motifs

**Figure 2.**
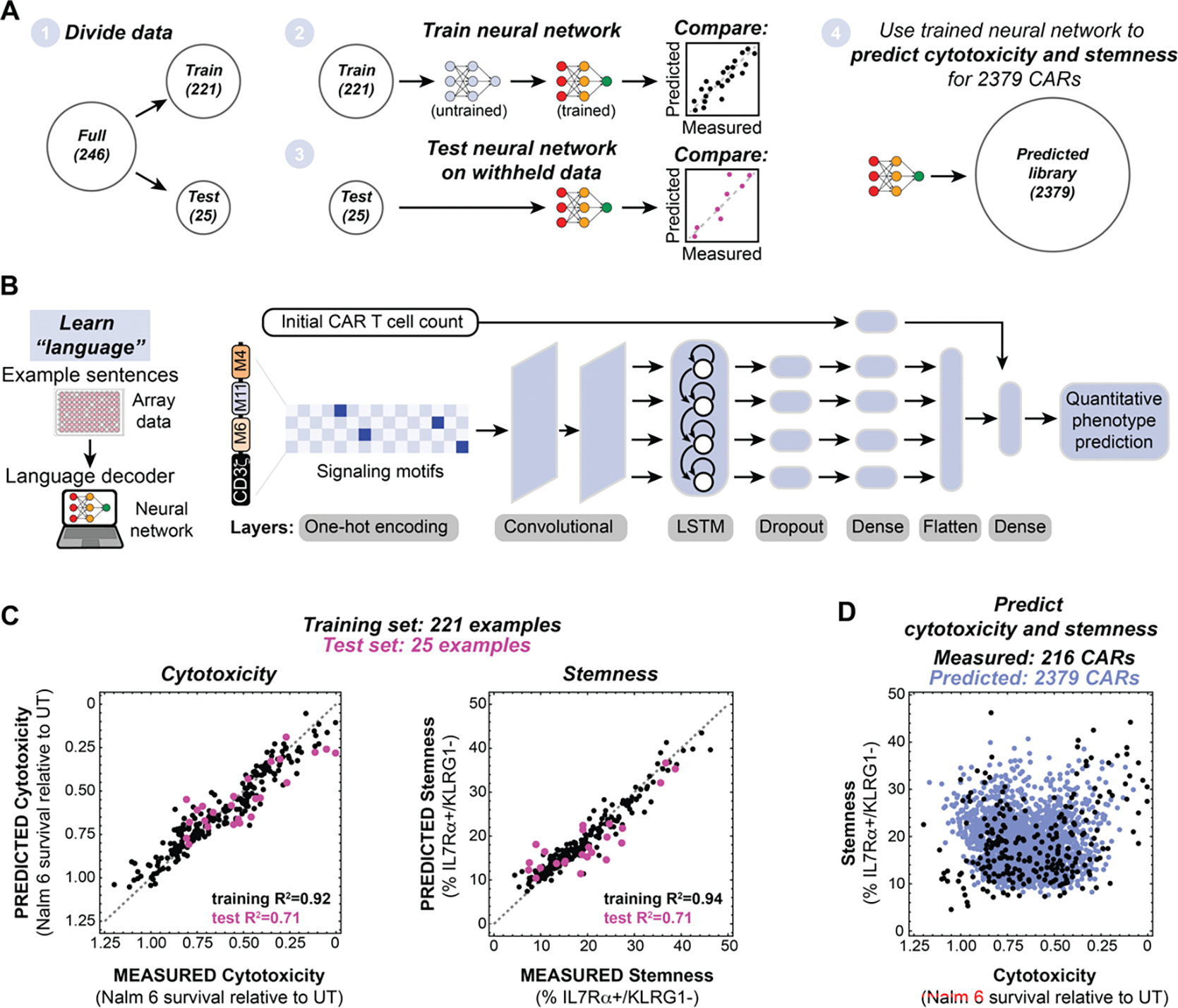
Neural networks decode the combinatorial language of signaling motifs to predict cytotoxicity and stemness of novel motif combinations. **A,** Array data were subdivided in datasets to train and test neural networks that were subsequently used to predict the cytotoxicity and stemness of 2379 CARs. **B**, Schematic of neural network used to predict CAR T cell phenotype. **C**, Neural networks trained on array data predict the cytotoxicity and stemness of CARs in the training sets (black) and the withheld test sets (pink). The root mean squared error (RMSE) for the cytotoxicity training set is 0.07579 and the RMSE for the cytotoxicity test set is 0.1327. RMSE for the stemness training set is 2.2038 and the RMSE for the stemness test set is 4.7941. **D,** Trained neural networks were used to predict the cytotoxicity and stemness of 2379 CARs containing 1–3 variable signaling motifs. Predictions represent the mean for n=10 neural networks with different hyperparameters.

The diverse cytotoxicity and stemness profiles observed in our arrayed screen are consistent with a complex relationship between signaling motif combinations and arrangement, and resulting T cell phenotypes. We sought to leverage the combinatorial nature of the costimulatory domain library by using machine learning to decode the “language” of signaling motifs that relates motif combinations to cytotoxicity and stemness outputs. We separated the arrayed screen data into a training set (221 examples) and a test set (25 examples). We then used these data sets to train several machine learning algorithms to predict cytotoxicity and stemness based on costimulatory domain identity and arrangement (Fig. 2A and Fig. S3). Neural networks (Fig. 2B) were best able to recapitulate the measured phenotypes in the training data (Fig. 2C) and to effectively predict the phenotypes in the test set (Fig. 2D). For both cytotoxicity and stemness training and test sets, the neural network was able to capture much of the relationship between signaling motif composition and phenotype, with R^2^ values of approximately 0.7 to 0.9.

The trained neural networks then allowed us to predict the CAR T cell cytotoxicity and stemness that would result from each of the 2379 motif combinations in the full combinatorial library (Fig. 2D), including those that were not part of the smaller arrayed screen. These simulated 2379 CARs sample the entire combinatorial space of the library, providing a dataset from which we extracted design rules. We analyzed: i) the overall contribution of each motif to a particular phenotype (without regard to combinatorial context); ii) identification of pairwise motif combinations that promote particular phenotypes, and iii) positional dependence of motifs.

### Distribution analysis summarizes the effects of signaling motifs, motif combinations, and motif positions on CAR T cell phenotype

**Figure 3.**
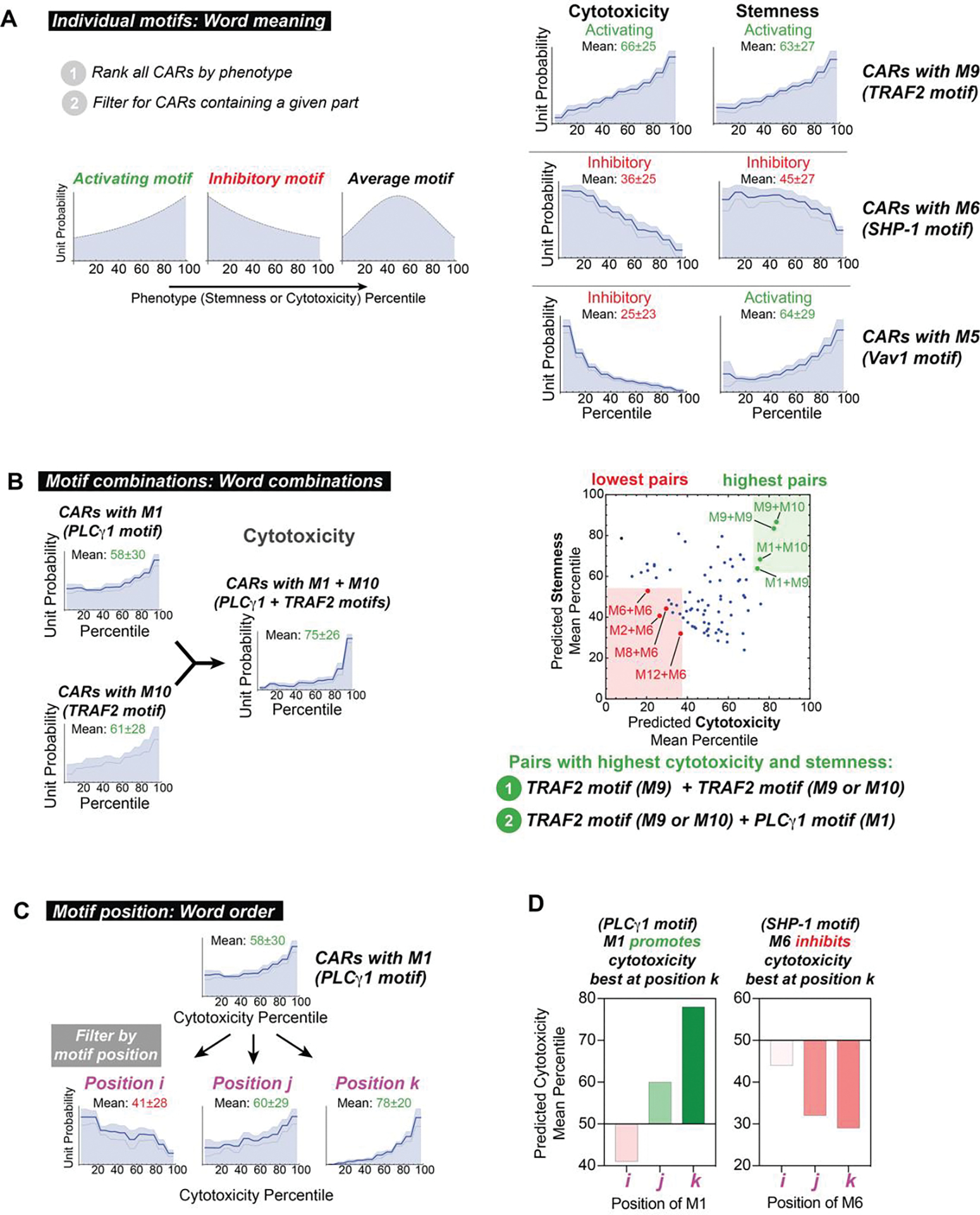
Distribution analysis quantifies elements of linear motif language to extract design parameters for signaling domains. **A**, The distribution of library parts throughout CARs in the ranked library reflects effects of signaling motifs on phenotype. Activating motifs are found in CARs with higher rank and inhibitory motifs are found in CARs with a lower rank. The three lines within the distributions represent mean predictions ± s.e.m. calculated from n=10 neural networks. **B**, CARs containing pairs of motifs that recruit TRAFs (M9 and M10) or PLCγ1 (M1) promote high cytotoxicity and stemness. Pairs promoting the highest and lowest cytotoxicity and stemness were determined by taking the sum of the mean percentile for each phenotype. **C**, Cytotoxicity percentile distributions for CARs containing M1 at various positions demonstrate that effects of signaling motifs on phenotype are position-dependent. **D**, Position-dependence of signaling motifs is quantified by calculating the mean of percentile distributions. M1 is predicted to promote cytotoxicity best at position k, while M6 is predicted to inhibit cytotoxicity best at position k.

To assess the overall contribution of individual motifs, we ranked all the CARs in our library by neural network-predicted cytotoxicity and stemness and then assessed whether motifs were enriched in the strong or weak ends of the phenotypic distribution (Fig. 3A, S4). If a motif is generally activating for a phenotype, then it is expected to be more common in highly ranked CARs; if a motif is inhibitory it is expected to be more common in poorly ranked CARs. Although the effects observed in this distribution analysis depend on other motifs in the CAR and the position of the motif in question, the distributions are informative of the overall effect each motif has in the context of the library. An analogous distribution analysis was also done on the pooled screening proliferation data (Fig. S5).

This distribution analysis highlighted several effective motifs that have activating and inhibitory roles. For example, motif M9 is the PQVE motif (from cluster of differentiation 40 (CD40)), which binds TRAF2, and is associated with T cell activation and function (24, 25). Accordingly, M9 is enriched in CARs with high cytotoxicity (mean 66^th^ percentile) and high stemness (mean 63^rd^ percentile), indicating that overall it promotes both of these phenotypes. In a contrasting example, M6 (from LAIR1) recruits the phosphatase SHP-1, an inhibitor of T cell activation. Accordingly, M6 is enriched in CARs with low cytotoxicity (mean 36^th^ percentile) and low stemness (mean 45^th^ percentile), indicative of inhibition of both phenotypes. Some motifs can activate one phenotype and inhibit another: M5, which binds Vav1, is unrepresented in CARs with high cytotoxicity (mean 25^th^ percentile), but overrepresented in CARs with high stemness (mean 64^th^ percentile). Thus, Vav1 signaling appears to promote stemness, while inhibiting killing. The quantified effects of all individual motifs on cytotoxicity and stemness are shown in the heatmap in Fig. S4. The TRAF binding motifs (M9 and M10) are among the best at promoting both cytotoxicity and stemness.

We anticipated that phenotypes would be highly dependent on motif combinations, as different downstream signaling pathways could be either complementary, redundant, or competing. To examine motif pairs that favored particular phenotypes, we examined the occurrence of each possible pair (without regard to order) in the ranked distribution. Several specific motif pairs appear to promote both cytotoxicity and stemness when they occur in combination within a costimulatory domain. For example, M1 (PLCγ1) and M10 (TRAF) were each activating with respect to cytotoxicity (means: 58^th^ and 60^th^), but the M1+M10 motif pair was even more strongly activating (mean: 75^th^ percentile). The predicted mean cytotoxicity and stemness percentiles for all 144 pairs of motifs M1 to M12 are shown in Fig. 3B. The motif pairs M1+M10, M1+M9, M9+M9, and M9+M10 were best at promoting cytotoxicity and stemness. These pairs all demonstrate that TRAF-binding motifs (M9 and M10) work well in tandem, as well as in combination with the motif that recruits PLCγ1 (M1) whose signaling activates nuclear factor kappa B (NFκB). Thus these pathways may serve complementary roles in these phenotypes. A number of motif pairs strongly inhibited cytotoxicity and stemness. All four motif pairs with the lowest cytotoxicity and stemness contain M6 which binds the inhibitory phosphatase SHP-1.

The phenotype of CAR T cells was highly dependent on the position of a motif within the costimulatory domain (Fig. S4B). For example, M1 (PLCγ1) showed strong cytotoxicity when in positions k or j, and weak cytotoxicity in position i (Fig. 3C). M9 (TRAF) and M10 (TRAF) showed optimal cytotoxicity and stemness when in positions i and j. This is consistent with the experimental observation that TRAF-binding parts M9 and M10 followed by M1 (in N- to C-terminal order) promote the most cytotoxicity and stemness (M1 followed by M9 or M10 does not (Fig. S2E). These results indicate that shuffling motif position is an approach for calibrating phenotype.

**Figure 4.**
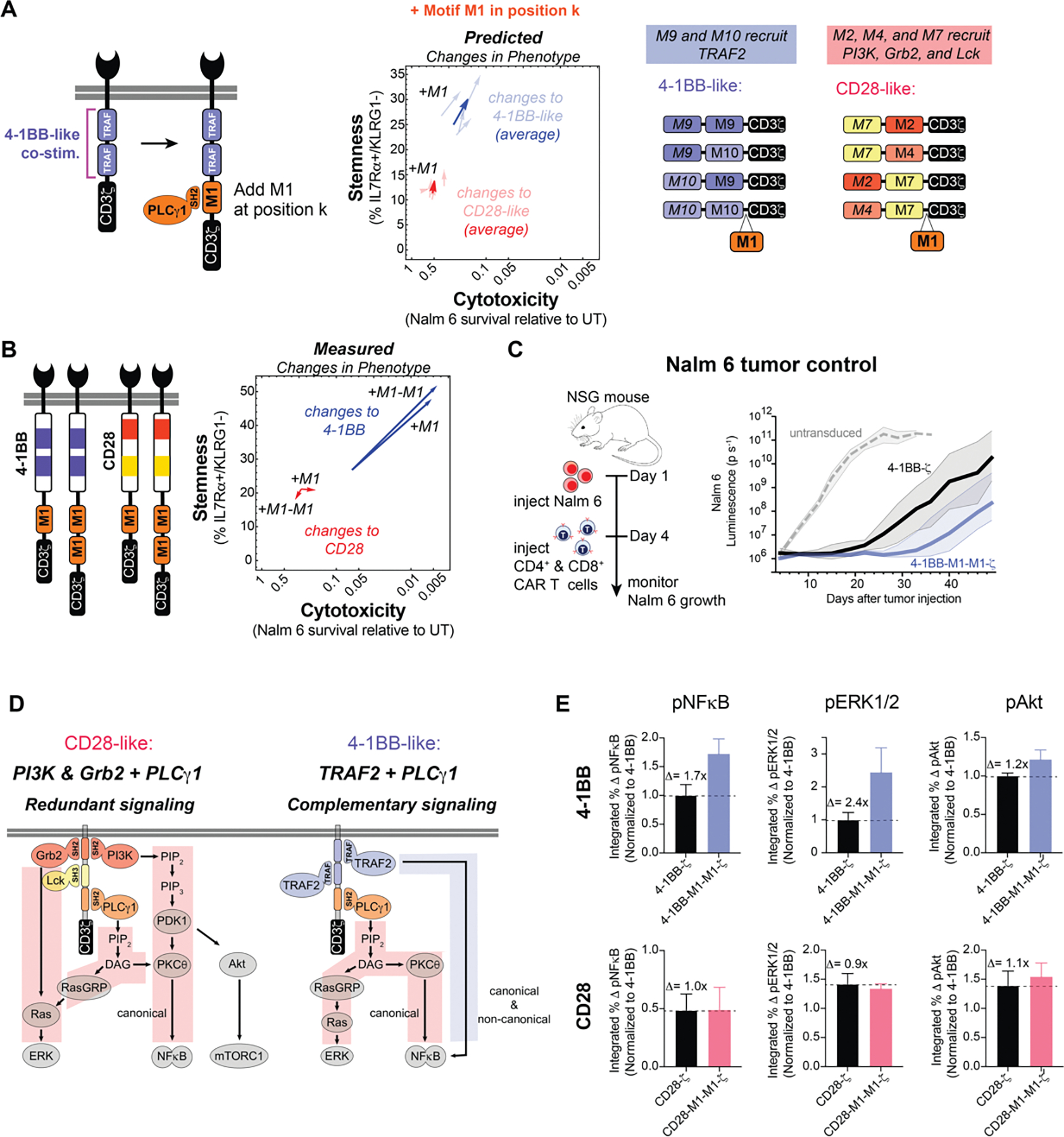
Neural networks accurately predict that PLCγ1 binding motifs improve the cytotoxicity and stemness of 4-1BB-ζ but not CD28-ζ. **A**, Library parts that share consensus signaling motifs with 4-1BB and CD28 costimulatory domains were used to predict the effect of adding at M1 to 4-1BB and CD28. **B**, Addition of 1 or 2 copies of M1 improved *in vitro* cytotoxicity and stemness of 4-1BB-ζ but not CD28-ζ. CAR T cell cytotoxicity and stemness were assessed after 4 pulses of Nalm 6 cells. Data are mean of *n* = 3–5 replicates. **C**, CD4+ and CD8+ CAR T cells were sorted for CAR expression 6 days after activation (1 day after Dynabead removal), and injected into mice 10 days later. NSG mice were injected intravenously with 0.5 × 106 Nalm 6 cells, and then injected intravenously with 3 × 106 CAR+ T cells on day 4. CAR T cells with 4-1BB-M1-M1-ζ showed improved early tumor control relative to 4-1BB-ζ. Traces in **C** are median luminescence ± s.e.m. confidence interval. **D**, Costimulatory PLCγ1 signaling is redundant to signaling provided by PI3K and Grb2, but complementary to TRAF signaling. Addition of M1 to 4-1BB-ζ induced modest changes in Akt phosphorylation—which is not downstream of PLCγ1 signaling—relative to the changes in ERK1/2 and NFκB phosphorylation—which are downstream of PLCγ1 signaling. **E**, CAR T cells were pulsed once with Nalm 6 leukemia cells and fixed at various timepoints from 0 to 60 minutes. Phosphorylation of ERK1 and ERK2, NFkB, and Akt was assessed by flow cytometry. Data for **E** are mean and standard deviation of n = 3 replicates. p, phosphorylated.

The above distribution analysis quantifies elements of motif language, capturing the effects of motifs (word meaning), motif pairs (word combinations), and motif position (word order) on phenotype. The analysis also yields design rules that can inform combinations and arrangements of motifs capable of producing a desired cell fate. For example, a synthetic costimulatory domain that contains one or more TRAF binding motifs (M9 or M10) followed by a PLCγ1 (M1) motif appears to be effective at promoting both cytotoxicity and stemness (Fig. 4A). Although tandem TRAF binding motifs occur in the naturally evolved 4-1BB receptor (26) (Fig. S6A), the combination of TRAF and PLCγ1 motifs is not found in natural characterized immune receptors. Thus, we tested whether adding PLCγ1 (M1) motifs to 4-1BB-like domains could improve phenotypes induced by CARs. Moreover, we also wanted to determine if adding M1 might be a general strategy to improve the efficacy of other costimulatory domains, such as CD28.

### Neural networks predict addition of M1 enhances the cytotoxicity and memory potential of 4-1BB-ζ but not CD28-ζ

We examined the neural network-predicted library to predict the effects of adding the M1 motif to CD28-like and 4-1BB-like synthetic costimulatory domains (library members whose signaling motifs shared the overall configuration of natural signaling motifs in CD28 and 4-1BB) (Fig. 4A). The 4-1BB-like costimulatory domains were predicted by the neural network model to show increased cytotoxicity and stemness, consistent with experimental observations. In contrast, addition of M1 motifs to CD28-like costimulatory domains was not predicted to enhance cytotoxicity or stemness.

To experimentally test this, we synthesized derivatives of the 4-1BB and CD28 costimulatory domains with 1 or 2 copies of the M1 motif added to the C-terminus, and tested the effects of these costimulatory domains on killing of Nalm 6 and maintenance of T cell stemness (Fig. 4B). Consistent with predictions, 4-1BB showed significantly enhanced cytotoxicity and stemness upon addition of M1, while CD28 showed almost no change. Significantly, in addition to predicted in vitro changes, the 4-1BB-M1-M1-ζ CAR construct showed improved efficacy in a Nalm 6 tumor Nod scid gamma (NSG) mouse model (Fig. 4C and Fig. S6). Relative to standard 4-1BB CAR T cells, the 4-1BB-M1-M1-ζ CAR T cells delayed the growth of Nalm 6 tumor cells for an additional two weeks, in agreement with the predictions from the library and neural network model.

Why might a PLCγ1 motif improve T cell phenotype in combination with the 4-1BB domain (TRAF motifs) but not in the context of the CD28 domain (PI3K, Grb2, Lck motifs)? PLCγ1 catalyzes the production of diacylglycerol (DAG) from phosphatidylinositol 4,5-bisphosphate (PIP_2_), which activates Ras guanyl-releasing protein (RasGRP) and protein kinase C theta (PKCθ), subsequently activating the extracellular signal-regulated kinases (ERK1 and 2) and NFκB. This signaling is similar and possibly redundant to that of PI3K and Grb2, which also activate RasGRP and PKCθ. TRAF signaling, however, does not activate RasGRP or PKCθ, such that PLCγ1 and TRAF signaling are more likely to be complementary (Fig. 4D). We experimentally characterized the 4-1BB-M1-M1-ζ CAR construct (compared to standard 4-1BB-ζ CAR) by measuring the kinetics of phosphorylation of protein kinase B (Akt), ERK1 and 2, and NFκB after stimulation by Nalm 6 (Fig. 4E and Fig. S7A). The addition of the M1 motifs increased phosphorylation of ERK1 and 2 (1.7-fold) and NFκB (2.4-fold), both of which depend on activation of PLCγ1. Phosphorylation of Akt, which is not dependent on PLCγ1, showed only a 1.2-fold increase. The observed increase in activation of NFκB and ERK1 and 2 supports the hypothesis that PLCγ1 signaling is complementary to TRAF signaling and is consistent with importance of NFκB activation for the maintenance of CD8+ T cell memory (27). In contrast, no significant increase in activation of NFκB and ERK1 and 2 was observed for a CAR in which the PLCγ1 motif was appended to the CD28 costimulatory domain. We observed little additional activation-induced increase in PLCγ1 phosphorylation in the cells bearing the 4-1BB-M1-M1-ζ CAR (Fig. S7C), suggesting that M1 may enhance signaling by altering the precise spatial organization of PLCγ1 binding sites (28) or by promoting PLCγ1-dependent LAT clustering (29).

## CONCLUSIONS

In conclusion, we find that signaling motif libraries and machine learning can be combined to elucidate rules of CAR costimulatory signaling and to guide the design of non-natural costimulatory domains with improved phenotypes, both in vitro and in vivo. Costimulatory signaling modulates the outcome of CAR T cell activation, making costimulatory domains attractive engineering targets for customizing or improving cell therapies. Thus far, costimulatory domain engineering has mostly been limited to the addition of intact natural domains such as those from 4-1BB, CD28, or the interleukin-2 receptor beta subunit (IL2Rβ), effectively using naturally occurring signaling sentences (motif combinations). We used motifs from receptors as words to generate thousands of different signaling sentences that drove T cells to distinct cell fates, potentially yielding more diverse and nuanced phenotypic meaning. Augmenting experimental analysis of a subset of receptors with neural network analysis allowed us to explore a larger region of combinatorial motif space. In particular, we identified the non-natural combination of TRAF- and PLCγ1-binding motifs that may be useful in CAR T cell therapies. With an arrayed screen of several hundred receptors and machine learning, we identified basic elements of signaling motif language and extracted design rules that relate motif combinations to cell fate. This represents a step toward forward engineering receptors with desired properties. Similar screening approaches with other CARs and target cancer cells are needed to determine the optimal signaling domains for each CAR and tumor type. Libraries may also be of use in identifying combinations of binding, hinge, linker, transmembrane, and signaling domains that produce optimal T cell function, and assessing the safety and toxicity of such combinations. Exploration of these larger libraries may benefit from machine learning due to the size and complexity of the combinatorial space. Machine learning-augmented screens of this type might be used to engineer many other classes of receptors for biological research and cell therapy applications involving cellular processes controlled by combinations of signaling motifs.

## Supplementary Material

S1

S3

S2

4

## Data Availability

Reagents are available from the corresponding author upon request from the authors. The pHR plasmid containing the anti-CD19 CAR and plasmids containing DNA for the 13 motifs used in the combinatorial library will be available from Addgene. Array screening data are available in supplemental information. Machine learning data analysis codes are available at https://github.com/CCCofficial/combinatorial_signaling_motif_libraries.

## References

[1] Barrett DM, Singh N, Porter DL, Grupp SA, June CH, Chimeric antigen receptor therapy for cancer. Annu Rev Med 65, 333–347 (2014). 24274181 10.1146/annurev-med-060512-150254 PMC4120077

[2] Irving BA, Weiss A, The cytoplasmic domain of the T cell receptor zeta chain is sufficient to couple to receptor-associated signal transduction pathways. Cell 64, 891–901 (1991). 1705867 10.1016/0092-8674(91)90314-o

[3] Romeo C, Seed B, Cellular immunity to HIV activated by CD4 fused to T cell or Fc receptor polypeptides. Cell 64, 1037–1046 (1991). 1900456 10.1016/0092-8674(91)90327-u

[4] Letourneur F, Klausner RD, T-cell and basophil activation through the cytoplasmic tail of T-cell-receptor zeta family proteins. Proc Natl Acad Sci U S A 88, 8905–8909 (1991). 1833767 10.1073/pnas.88.20.8905 PMC52619

[5] Krause A , Antigen-dependent CD28 Signaling Selectively Enhances Survival and Proliferation in Genetically Modified Activated Human Primary T Lymphocytes. Journal of Experimental Medicine 188, 619–626 (1998). 9705944 10.1084/jem.188.4.619 PMC2213361

[6] Finney HM, Lawson ADG, Bebbington CR, Weir ANC, Chimeric Receptors Providing Both Primary and Costimulatory Signaling in T Cells from a Single Gene Product. The Journal of Immunology 161, 2791–2797 (1998). 9743337

[7] Imai C , Chimeric receptors with 4-1BB signaling capacity provoke potent cytotoxicity against acute lymphoblastic leukemia. Leukemia 18, 676–684 (2004). 14961035 10.1038/sj.leu.2403302

[8] Ramos CA , In Vivo Fate and Activity of Second- versus Third-Generation CD19-Specific CAR-T Cells in B Cell Non-Hodgkin’s Lymphomas. Mol Ther 26, 2727–2737 (2018). 30309819 10.1016/j.ymthe.2018.09.009 PMC6277484

[9] Enblad G , A Phase I/IIa Trial Using CD19-Targeted Third-Generation CAR T Cells for Lymphoma and Leukemia. Clin Cancer Res 24, 6185–6194 (2018). 30097433 10.1158/1078-0432.CCR-18-0426

[10] Kagoya Y , A novel chimeric antigen receptor containing a JAK-STAT signaling domain mediates superior antitumor effects. Nat Med 24, 352–359 (2018). 29400710 10.1038/nm.4478 PMC5839992

[11] Koch CA, Anderson D, Moran MF, Ellis C, Pawson T, SH2 and SH3 Domains: Elements that Control Interactions of Cytoplasmic Signaling Proteins. Science 252, 668–674 (1991). 1708916 10.1126/science.1708916

[12] Sudol M, From Src Homology domains to other signaling modules: proposal of the `protein recognition code’. Oncogene 17, 1469–1474 (1998). 9779993 10.1038/sj.onc.1202182

[13] Kawalekar OU , Distinct Signaling of Coreceptors Regulates Specific Metabolism Pathways and Impacts Memory Development in CAR T Cells. Immunity 44, 380–390 (2016). 26885860 10.1016/j.immuni.2016.01.021 PMC13498396

[14] Gordon KS , Screening for CD19-specific chimaeric antigen receptors with enhanced signalling via a barcoded library of intracellular domains. Nat Biomed Eng 6, 855–866 (2022). 35710755 10.1038/s41551-022-00896-0 PMC9389442

[15] Goodman DB , Pooled screening of CAR T cells identifies diverse immune signaling domains for next-generation immunotherapies. Science Translational Medicine 14, eabm1463 (2022). 36350984 10.1126/scitranslmed.abm1463 PMC9939256

[16] Castellanos-Rueda R , speedingCARs: accelerating the engineering of CAR T cells by signaling domain shuffling and single-cell sequencing. Nat Commun 13, 6555 (2022). 36323661 10.1038/s41467-022-34141-8 PMC9630321

[17] Dinkel H , ELM--the database of eukaryotic linear motifs. Nucleic Acids Res 40, D242–251 (2012). 22110040 10.1093/nar/gkr1064 PMC3245074

[18] Houtman JCD , Binding Specificity of Multiprotein Signaling Complexes Is Determined by Both Cooperative Interactions and Affinity Preferences. Biochemistry 43, 4170–4178 (2004). 15065860 10.1021/bi0357311

[19] Xu M, Zhao R, Zhao ZJ, Identification and characterization of leukocyte-associated Ig-like receptor-1 as a major anchor protein of tyrosine phosphatase SHP-1 in hematopoietic cells. J Biol Chem 275, 17440–17446 (2000). 10764762 10.1074/jbc.M001313200

[20] Borsa M , Modulation of asymmetric cell division as a mechanism to boost CD8+ T cell memory. Science Immunology 4, eaav1730 (2019). 30979796 10.1126/sciimmunol.aav1730

[21] Herndler-Brandstetter D , KLRG1(+) Effector CD8(+) T Cells Lose KLRG1, Differentiate into All Memory T Cell Lineages, and Convey Enhanced Protective Immunity. Immunity 48, 716–729 e718 (2018). 29625895 10.1016/j.immuni.2018.03.015 PMC6465538

[22] Wu H , Expression of KLRG1 and CD127 defines distinct CD8(+) subsets that differentially impact patient outcome in follicular lymphoma. J Immunother Cancer 9, (2021). 10.1136/jitc-2021-002662 PMC8258669 34226281

[23] Remmerswaal EBM , Expression of IL-7Ralpha and KLRG1 defines functionally distinct CD8(+) T-cell populations in humans. Eur J Immunol 49, 694–708 (2019). 30883723 10.1002/eji.201847897 PMC6593687

[24] Schoenberger SP, Toes REM, van der Voort EIH, Offringa R, Melief CJM, T-cell help for cytotoxic T lymphocytes is mediated by CD40–CD40L interactions. Nature 393, 480–483 (1998). 9624005 10.1038/31002

[25] McWhirter SM , Crystallographic analysis of CD40 recognition and signaling by human TRAF2. Proceedings of the National Academy of Sciences of the United States of America 96, 8408–8413 (1999). 10411888 10.1073/pnas.96.15.8408 PMC17529

[26] Park HH, Structure of TRAF Family: Current Understanding of Receptor Recognition. Front Immunol 9, 1999 (2018). 30214450 10.3389/fimmu.2018.01999 PMC6125299

[27] Knudson KM , NFkappaB-Pim-1-Eomesodermin axis is critical for maintaining CD8 T-cell memory quality. Proc Natl Acad Sci U S A 114, E1659–E1667 (2017). 28193872 10.1073/pnas.1608448114 PMC5338529

[28] Basant A, Way M, The relative binding position of Nck and Grb2 adaptors impacts actin-based motility of Vaccinia virus. Elife 11, (2022). 10.7554/eLife.74655 PMC9333988 35796545

[29] Zeng L, Palaia I, Saric A, Su X, PLCgamma1 promotes phase separation of T cell signaling components. J Cell Biol 220, (2021). 10.1083/jcb.202009154 PMC8094118 33929486

[30] Jung Y, Hu J, A K-fold averaging cross-validation procedure. Journal of Nonparametric Statistics 27, 167–179 (2015). 27630515 10.1080/10485252.2015.1010532 PMC5019184

[31] Géron A, Hands-on machine learning with Scikit-Learn, Keras, and TensorFlow: Concepts, tools, and techniques to build intelligent systems. (O’Reilly Media, 2019).

[32] Wang S , Massive computational acceleration by using neural networks to emulate mechanism-based biological models. Nat Commun 10, 4354 (2019). 31554788 10.1038/s41467-019-12342-y PMC6761138

