# Supplementary material for: Decoding CAR T cell phenotype using combinatorial signaling motif libraries and machine learning
Source: Science. Author manuscript; Available in PMC 2023 Mar 20. (PMC10026561; doi:10.1126/science.abq0225)
Supplement: 4 [file NIHMS1871220-supplement-4.pdf]

Supplementary Materials for

Engineering CAR T cell phenotype using combinatorial signaling motif libraries and machine learning

Kyle G. Daniels[1], Shangying Wang[2,3], Milos S. Simic[1], Hersh K. Bhargava[1], Sara Capponi[2,3], Yurie Tonai[1], Wei Yu[1], Simone Bianco[2,3, †*], Wendell A. Lim[1*]

Correspondence to: sbianco@altoslabs.com; wendell.lim@ucsf.edu

This PDF file includes:

Materials and Methods
Figs. S1 to S7
Tables S1 to S2
Captions for Data S1

Other Supplementary Materials for this manuscript include the following:

Data S1

Materials and Methods

Viral vector construction

Codon-optimized DNAs encoding the variable library parts were codon optimized for expression in human cells using ThermoFisher GeneArt's website tool and synthesized by ThermoFisher GeneArt. A pHR lentiviral vector containing an SFFV promoter followed by DNA encoding the aCD19 scFv and the CD8a hinge and transmembrane domain was BamHI restriction digested. DNA encoding a BamHI cut site followed by CD3z-P2A-EGFP was subcloned into the digested pHR-aCD19-(BamHI)-CD3z-P2A-EGFP vector to create library backbone. DNA encoding library parts, as well as DNA encoding 4-1BB, CD28, and ICOS, was subcloned into the library backbone and the cloning product was again BamHI digested. This was repeated to create vectors with 3 variable library parts. All constructs were built via in-fusion cloning (Clontech #ST0345) and sequence verified before use. Amino acid sequences for library parts were as follows. M1: DYHNPGYLVVLPDSTP, M2: EELDENYVPMNPNSPP, M3: EEGAPDYENLQELNHP, M4: LGSNQEEAYVTMSSFYQNQ, M5: LPMDTEVYESPFADPEEIR, M6: KPMAESITYAAVARHSAG, M7: LPTWSTPVQPMALIVLG, M8: PAPSIDRSTKPPLDRSL, M9: GSNTAAPVQETLHGCQ, M10: DDSLPHPQQATDDSGHES, M11: KAPHAKQEPQEINFPDDLP, M12: GSGPGSRPTAVEGLALGSS, M13: SAGSAGSAGSAGSAGSAG.

Cell lines

Nalm 6 cell lines were originally obtained from ATCC (CRL-3273) and were stably transduced with mCherry and firefly luciferase. Nalm 6 cell lines were cultured in RPMI-1640 + GlutaMAX (Gibco #72400-047) supplemented with 10% FBS (UCSF Cell Culture Facility).

Primary human T cell isolation and culture

Primary CD4+ and CD8+ T cells were isolated from blood of anonymous donors by negative selection using the Human CD4+ T cell isolation kit and Human CD8+ T cell isolation kit (STEMCELL Technologies #17952 and #17953). T cells were cryopreserved

in RPMI1640 (UCSF cell culture core) with 20% human AB serum (Valley Biomedical, #HP1022HI) and 10% DMSO. Upon thawing, T cells were cultured in human T cell medium (HTCM) consisting of X-VIVO 15 (Lonza #04-418Q), 5% Human AB serum, 1 mM 2-mercaptoethanol (Gibco #21985-023), and 10 mM neutralized N-acetyl L-Cysteine (Sigma-Aldrich #A9165) supplemented with 30 units/mL IL-2 (NCI BRB Preclinical Repository). Before co-culture with Nalm 6, T cells were transferred to HTCM without IL-2.

Lentiviral Transduction and Sorting of Human T Cells

Pantropic vesicular stomatitis virus G pseudotyped lentivirus was produced via transfection of LentiX 293T cells (Clontech #11131D) with a pHR'SIN:CSW transgene expression vector and the viral packaging plasmids pCMVdR8.91 and pMD2.G using FuGENE HD (Promega, #E2312). Primary T cells were thawed the same day and after 24 h in culture, were stimulated with Dynabeads Human T-Activator CD3/CD28 (Life Technologies #11131D) at 25 µL per 1 × 10^6 T cells. At 48 h (day 2), viral supernatant was harvested via centrifugation at 500 G for 5 min, and the primary T cells were exposed to the virus for 24 h in a 6-well plate (pooled screens) or in 96-well plates (arrayed screens). Dynabeads were removed at day 5 post-T cell stimulation. For pooled screens, GFP+ T cells were sorted on day 6 post-T cell stimulation with a FACSAria II. Assays were performed 10 days after removal of Dynabeads.

Arrayed Screening

CARs were constructed as described above in Viral Vector Construction. An additional pooled CAR sub-library was constructed with enriched concentration of DNA corresponding to M1, M4, M7, M9, and M10 on the basis of their high proliferation, degranulation, and memory formation in the pooled screening assay. Pooled CAR library DNA was used to transform 5-alpha F' I^q competent E. coli cells (New England BioLabs C2992H), which were then plated on LB/Carbenicillin. At 24 hours 384 colonies (288 from the unbiased library, and 96 from the high-performance sub-library) were picked and miniprepped, added to 96-well plates and sequence verified. Wells with failed sequencing results or unidentifiable sequences were removed from plates and the well contents were

replaced with duplicates of nearby wells, TE buffer (for empty well controls), or standard costimulatory domain (4-1BB, CD28) controls. CARs containing 4-1BB, CD28, and ICOS costimulatory domains and M1-M13 were left in place, but excluded from the analysis. One plate of the arrayed screen was tested in triplicate with three different T cell donors: once in the context of the four-plate arrayed screen, and twice independently to ensure that trends in the four-plate screen were representative and reproducible. Screening of neural network-inspired receptors was performed in triplicate technical replicates.

Primary human T cells transduced with CAR library constructs were mixed with Nalm 6 to reach 1×10^6 T cells per mL and 2×10^6 per mL Nalm 6 and centrifuged at 300g for 2 min. For day 3, 5, and 7 challenges with Nalm 6, 80 µL of co-cultured T cells and Nalm 6 were added to 120 µL of Nalm 6 at 2×10^6 per mL and centrifuged at 300g for 2 min.

For analysis of cell surface receptor expression, samples were centrifuged at 500 × g for 5 min and resuspended in a 50 µL volume with the appropriate antibodies diluted 1:50 in calcium-free magnesium-free PBS with 5% FBS and 5mM EDTA. After a 30-min incubation at room temperature, samples were washed twice calcium-free magnesium-free PBS with 5% FBS and 5mM EDTA. (FBS; UCSF Cell Culture Facility). Samples were analyzed for protein expression on a BD LSRII. Antibodies are as follows: APC Mouse anti-human KLRG1 clone SA231A2 (BioLegend #367716), BV421 Mouse anti-human IL7Rα clone HIL-7R-M21 (BD Biosciences #562436), BV786 Mouse anti-human CD62L clone SK11 (BD Biosciences #565311), AF700 Mouse anti-human CD45RA clone HI100 (BD Biosciences #560673), PE Mouse anti-human CD4 clone RPA-T4 (BD Biosciences #555347).

Pooled Screening

Primary human T cells transduced with pooled virus for the CAR library were mixed with Nalm 6 to reach 1×10^6 T cells per mL and 2×10^6 per mL Nalm 6. For day 3, 5, and 7 challenges with Nalm 6, co-cultured T cells and Nalm 6 were centrifuged at 400g for 4 min and resuspended at 1×10^6 per mL in 1/3 current HTCM and 2/3 fresh HTCM. Additional Nalm 6 were added at 2×10^6 per mL.

For extracellular staining, samples were centrifuged at 500g for 5 minutes and resuspended in FACS buffer with 1:50 PE anti-human CD4 antibody, 1:50 BV421 mouse anti-human CD45RA antibody, and 1:50 AF647 mouse anti-human CCR7 antibody. After a 30-min incubation at room temperature, samples were washed twice, and resuspended in FACS buffer. Samples were sorted on a BD FACS AriaII.

For analysis of degranulation on Day 9 Nalm 6 challenge, samples of $1x10^6$ per mL pooled CAR T cells with $2×10^6$ per mL Nalm 6 were centrifuged at 300 × g for 2 min in 96-well flat-bottom plates and incubated in HTCM with 1x Brefeldin-A/GolgiPlug (BDBiosciences #555029), 1x Monensin/GolgiStop (BD Biosciences #554724), and 1:50 APC anti-human CD107A antibody at 37C and 5% CO_2 for 5 hours. At 5 hours, samples were centrifuged at 300g for 2 minutes and supernatant was removed. Samples were resuspended in FACS buffer with 1x GolgiPlug, 1x GolgiStop, and 1:50 PE anti-human CD4 antibody. After a 30-min incubation at room temperature, samples were washed twice, and resuspended in FACS buffer. Samples were sorted on a BD FACS AriaII. Antibodies were as follows: PE Mouse anti-human CD4 clone RPA-T4 (BD Biosciences #555347), BV421 Mouse anti-human CD45RA clone HI100 (BD Biosciences #562885), AF647 Mouse anti-human CCR7 clone 150503 (BD Biosciences # 560816), APC Mouse anti-human CD107A clone H4A3 (Biosciences #641581).

Genomic DNA was extracted from sorted T cells using the Macherey-Nagel NucleoSpin Tissue XS kit (Takara #740901.250). DNA encoding the CAR costimulatory domain was amplified from the extracted genomic DNA using the forward primer 5'-TCGTCGGCAGCGTCAGATGTGTATAAGAGACAGNNNNNACTGGTTATCACCCTTTACTGC-3' (Integrated DNA Technologies) and reverse primer 5'-GTCTCGTGGGCTCGGAGATGTGTATAAGAGACAGNNNNNCTTGTAGGCGGGAGCAT-3' (Integrated DNA Technologies). Indexes were added to the amplified DNA using i5 and i7 primers from the Nextera XT Index Kit (Illumina # FC-131-1002). Indexed samples were loaded into a MiSeq Reagent Kit v3 600-cycle (Illumina # MS-102-3003) cartridge sequenced on a MiSeq (Illumina). Pooled screening assays were performed at least three

times using unique T cell donors and included data are representative. Reads for each CAR costimulatory domain construct were counted using software provided by Ian Webster at Zenysis Technologies.

Assessment of Akt, ERK1/2, and NFκB phosphorylation

For intracellular phospho-signaling analysis, T cells and Nalm 6 cells were plated at 1:2 ratio in HTCM and centrifuged at 400g for 3 minutes. Cells were plated and centrifuged at t= 0, 30, 45, 50, 55, and 59 min to obtain timepoints for approximately 60, 30, 15, 10, 5, and 1 minutes of T cell:Nalm 6 engagement. After the final sample was centrifuged, all samples were mixed 1:1 with prewarmed CytoFix Fixation Buffer (BDBiosciences #554655) and incubated at room temperature for 15 minutes. Samples were washed twice with FACS buffer, vigorously vortexed, and permeabilized with BD Phosflow Perm Buffer II (BDBiosciences #558050) by incubating overnight at -20C. Permeabilized samples were washed twice with FACS buffer and resuspended in 50 μL of FACS buffer with 1:50 anti-human phospho-NFκB antibody, 1:50 anti-human phospho-Akt antibody, and 1:50 anti-human phospho-ERK1/2 antibody. After a 60-minute incubation, samples were washed twice with FACS buffer and analyzed by flow cytometry on a BD LSRII. Antibodies are as follows: BV421 Mouse anti-human NFκB pS529 clone K10-895.12.50 (BD Biosciences #565446), PerCP-Cy5.5 Mouse anti-human ERK1/2 pT202/pY204 clone 20A (BD Biosciences #560115), PE-CF594 Mouse anti-human Akt pS473 clone M89-61 (BD Biosciences #562465), AF647 Mouse anti-human Akt pS473 clone M89-61 (BD Biosciences #560343).

Flow cytometry data were analyzed in FlowJo (BD) software to calculate mean fluorescence intensity (MFI) for pErk1/2, pNFκB, and pAkt channels. MFI kinetic traces were interpolated and integrated in *Mathematica* (Wolfram) to calculate the total change over 60 minutes in MFI for CAR T cell samples relative to the change in MFI of the untransduced control. Integrated changes in MFI were normalized to 4-1BB measurements to standardize experiments performed on different days.

Mice

All mouse experimental procedures were conducted according to Institutional Animal Care and Use Committee (IACUC)–approved protocols (AN183960-02A). The experiments were planned and independently performed by UCSF based on UCSF studies. Female immunocompromised NOD-SCID-*Il2rg*$^{-/-}$ (NSG) mice were obtained from UCSF breeding core. On Day 1, mice were inoculated with 0.5×10^6 Nalm 6 leukemia via tail vein injection. On Day 4, mice were injected with 3×10^6 T cells via tail vein injection. Leukemia progression was measured by bioluminescent imaging using the IVIS 100 (Xenogen) preclinical imaging system. Images were acquired 15 minutes following intraperitoneal (i.p.) injection with 150 mg/kg of D-luciferin (Gold Technology #LUCK-100). Display and adjustment of bioluminescence intensities was performed using the Living image 4.5.4 software (Perkin Elmer). Mice were humanely euthanized when IACUC-approved endpoint (hunching, neurological impairments such as circling, ataxia, paralysis, limping, head tilt, balance problems, seizures, tumor volume burden) was reached (10 mice per group).

Machine Learning

Data Preparation

For the arrayed data, in addition to the positional information of the combinational motifs, the initial CAR T cell number is a variable which affects the experimental output. Both are inputs of the machine learning algorithms. We randomly split D2 and use 90% for training and 10% for test, (we repeated this splitting process until duplicate motif combinations were found either exclusively in the training sets or exclusively in the test sets) ensuring all the motif combinations in the test data are different from those in the training.

We used a one-hot encoding to input motif combinations into the machine learning algorithms. Each motif position was described by a vector of fifteen 0s, and one 0 in each vector was replaced with a 1 corresponding to the absence of a motif (replace the first 0 with 1), the presence of a motif (replace the 0 equal to the part number + 1 with 1), or the presence of CD3z (replace the 15th 0 with 1). During synthesis of the 3-motif library, several CARs with 5 motifs were created. Rather than discard the data for these CARs,

we allowed up to 5 motif positions in the model, as well as CD3z, for a total of 6 vectors. This allows for inclusion of a small number of CARs that contained more than 3 motifs.

Machine learning Framework

In this work, we used a Convolutional Neural Networks (CNN), followed by a Long Short-Term Memory (LSTM) network together with fully connected layers. The code is implemented in both *Mathematica* (Wolfram) and Python 3.7.8 with TensorFlow v2.4.1, both of which produce nearly identical results. The Mathematica analysis is described below. The neural network uses the $A X B$ matrices as inputs and outputs one value corresponding to one of the phenotypes (cytotoxicity and stemness). Between the input and output layers, we have 2 convolutional layers, 1 LSTM layer, 1 dropout layer, and several fully connected layers. The convolutional layers detect spatial correlations in input data and the LSTM layer learns the long-term dependencies of the sequence data. We used dropout regularization to prevent over-fitting. The dropout layer connects to fully connected layers which are then flattened and catenated with the cell number input and connect to a dense layer. We used linear activation function to connect this dense layer and the final output layer. For training in Mathematica, we used mean squared error loss and ADAM optimization algorithm with automatic learning rate, and training over 200 iterations.

We also compared our methods with other widely used machine learning regression methods, such as k-nearest neighbor regression, linear regression, nearest neighbors, random forest regression, and gradient boosted regression. The CNN + LSTM neural network has the best performance and predictive power of the methods compared.

Selection of neural network Hyperparameters

We tuned the hyperparameters for layers in the neural networks to find optimal hyperparameters for the cytotoxicity and stemness datasets. The tuned hyperparameters include convolutional layers filters (10, 20, 50), kernel size (2, 3, 4, 5); LSTM layer units (2, 4, 8), dropout layer dropout rate (0, 0.1, 0.2), and fully connected layer units (6, 14, 64).

Hyperparameters were tuned as follows: We performed a grid search of hyperparameters and scored each parameter set by 10-fold cross validation of the training set. The best-performing 10 hyperparameter sets for each dataset (cytotoxicity or stemness) were selected using the K-fold averaging cross validation (ACV) method and used to train 10 neural networks whose outputs were then averaged (*30*). The trained neural networks were used to simulate the cytotoxicity and stemness for the 2379 combinations of 1, 2, or 3 variable motifs at a fixed initial cell count of 2000 cells (corresponding to 2000 CAR T cells in 40 μL of flow cytometry sample).

Hyperparameters for final neural networks are available in supplementary tables.

Ensemble Method

Due to the stochastic nature of network initialization and dropout, as well as the availability of a limited training set, every neural network is unique in terms of the parameterization of the network connections (*31, 32*). To mitigate the potential impact of this issue, we implemented an ensemble decision method to obtain consensus prediction from ten identical neural networks.

Distribution analysis

Distribution analysis was performed in *Mathematica* (Wolfram). CARs were sorted by proliferation (lowest enrichment to highest enrichment), cytotoxicity (highest Nalm 6 survival to lowest Nalm 6 survival), or stemness (lowest %IL7Rα+/KLRG1- to highest %IL7Rα+/KLRG1-) and assigned percentiles from 0 to 100. Individual parts or motif analysis was performed by selecting all CARs that contain a given part of interest. Pairs of parts or motifs analysis was performed by selecting all CARs that contain a given pair of parts. Position analysis was performed by selecting all CARs that contain a given part at a position of interest. Distributions for selected CARs were constructed using the HistogramDistribution functionality and smoothed by using the PDF (probability distribution function) functionality to calculate the probability from 2.5th percentile to 97.5th percentile in steps of 5. The mean and standard error of the mean for each distribution was calculated by repeating the above processing for each of 10 neural networks (for predicted array screen data) or for experimental replicates (pooled screen data).

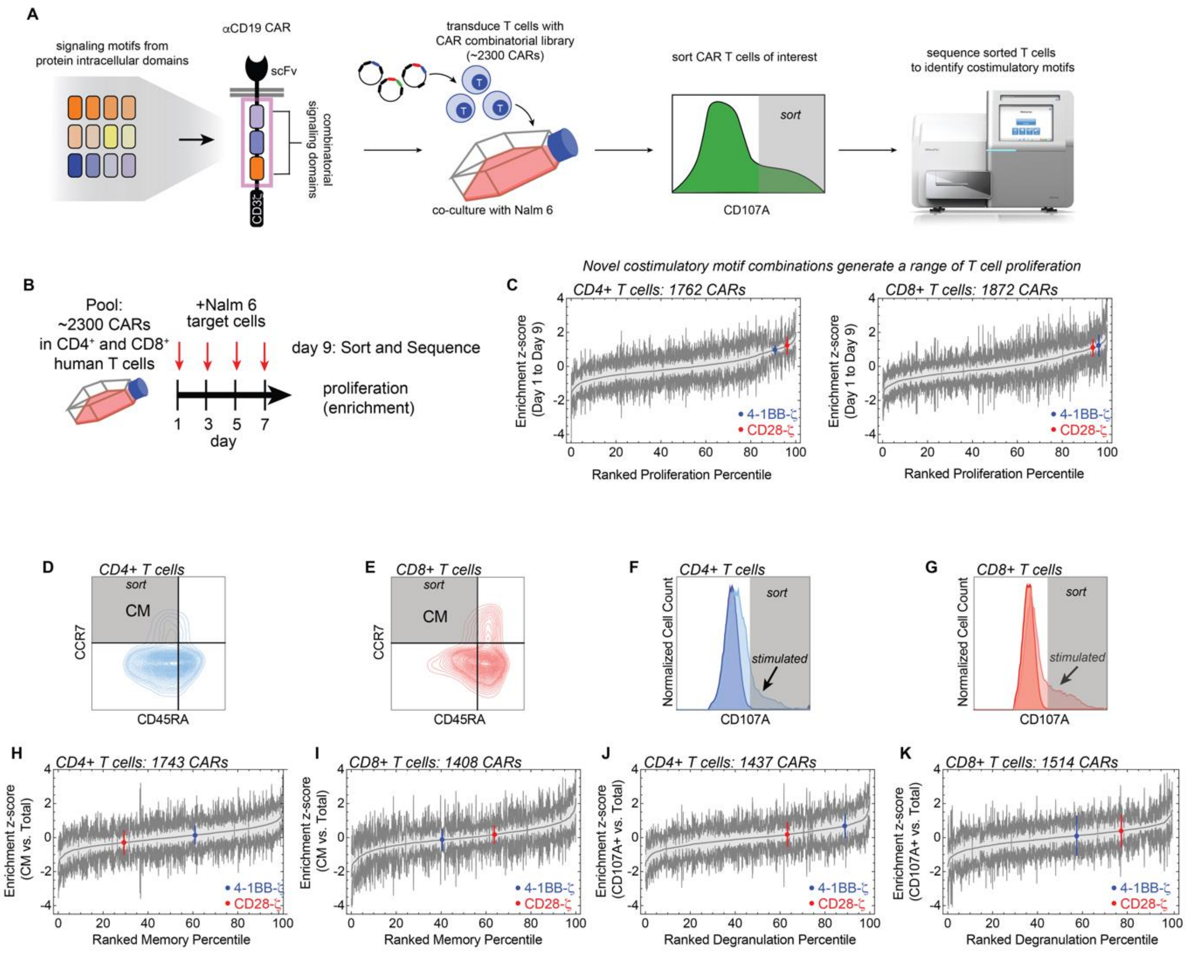

Figure S1. CARs with novel signaling motif combinations generate diverse T cell outputs of proliferation, memory formation, and degranulation in a pooled screen. **A**, Workflow for pooled screening of pooled combinatorial CAR library. **B**, Timeline for pooled combinatorial CAR library screen. **C**, CAR T cell proliferation calculated by Log2 fold change in CAR construct frequency 9 days after initial stimulation relative to the starting populations indicates the constructs in the library promote differing degrees of T cell proliferation. **D-G**, Select populations of central memory cells and degranulating cells were isolated by FACS according to the gates shown. Isolated cells were later sequenced. **H-K**, CAR T cell memory formation and degranulation were calculated by Log2 fold change in CAR construct frequency in FACS-isolated select populations on day 9 relative to total populations on day 9. Constructs in the library promote differing degrees of central memory formation and degranulation. All enrichment plot data are mean ± s.e.m. of *n* = 3 replicates.

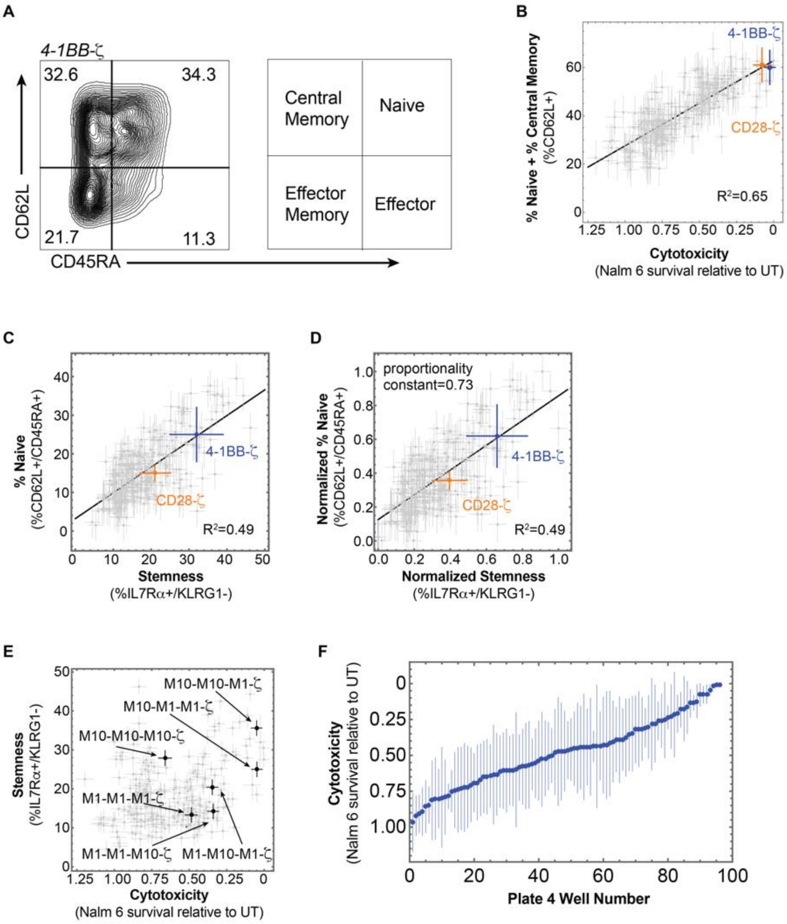

Figure S2. CAR costimulatory domains with novel signaling motif combinations generate diverse cell fates. A, CAR T cells with novel signaling motif combinations generate a broad range of naïve + central memory populations (quantified by CD62L expression). **B,** CD62L expression is positively correlated with cytotoxicity (r^2=0.65). Errors for Nalm 6 survival, and %CD62L+ population in **B** were estimated by calculating the average s.e.m. for 7 CAR constructs in the array with internal replicates. **C,** Stemness of CAR T cells is nearly proportional to the frequency of the naïve population. **D,** The best fit line for normalized stemness versus percent naïve yields a proportionality constant of 0.7. Black lines in **B**, **C,** and **D** represent best fit of the data to a line. **E,** CARs containing M1 and M10 generate high cytotoxicity and stemness when combined such that M1 is distal from the membrane, but generate reduced cytotoxicity and stemness when not combined or when M1 is not distal from the membrane. **F**, One 96-well plate in the arrayed screen was tested for cytotoxicity using three donors to assess donor-to-donor variability.

Error bars represent standard deviation among the three replicates. Variability in cytotoxicity also reflects variability in T cell transduction efficiency.

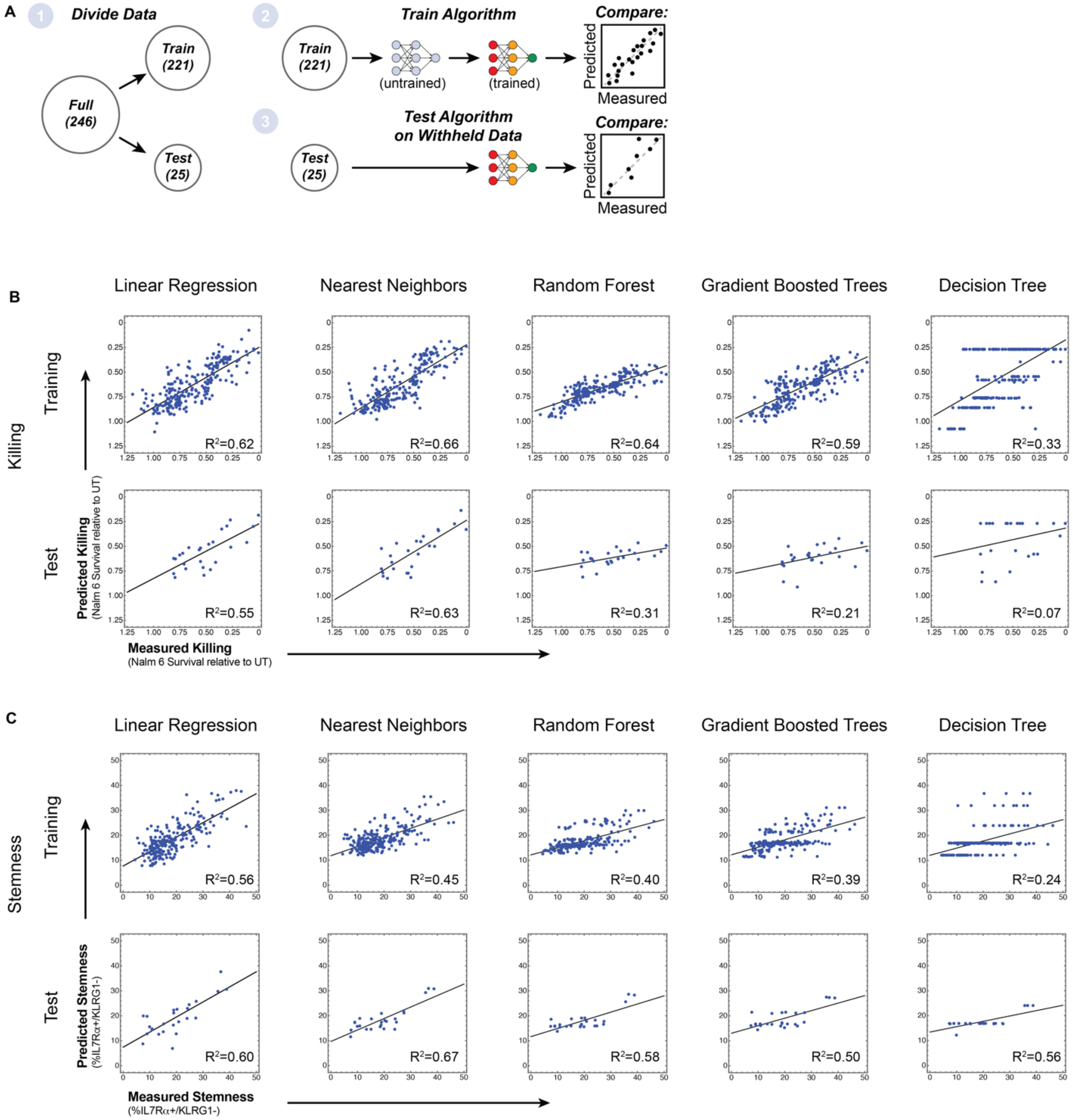

Figure S3. Several common machine learning algorithms fail to predict CAR T cell phenotype. A, Array data were subdivided in datasets to train and test various machine learning algorithms. **B-C,** Linear regression, nearest neighbors, random forest, gradient boosted trees, and decision tree algorithms were used to predict cytotoxicity (**B**) and stemness (**C**) resulting from various combinations of library parts.

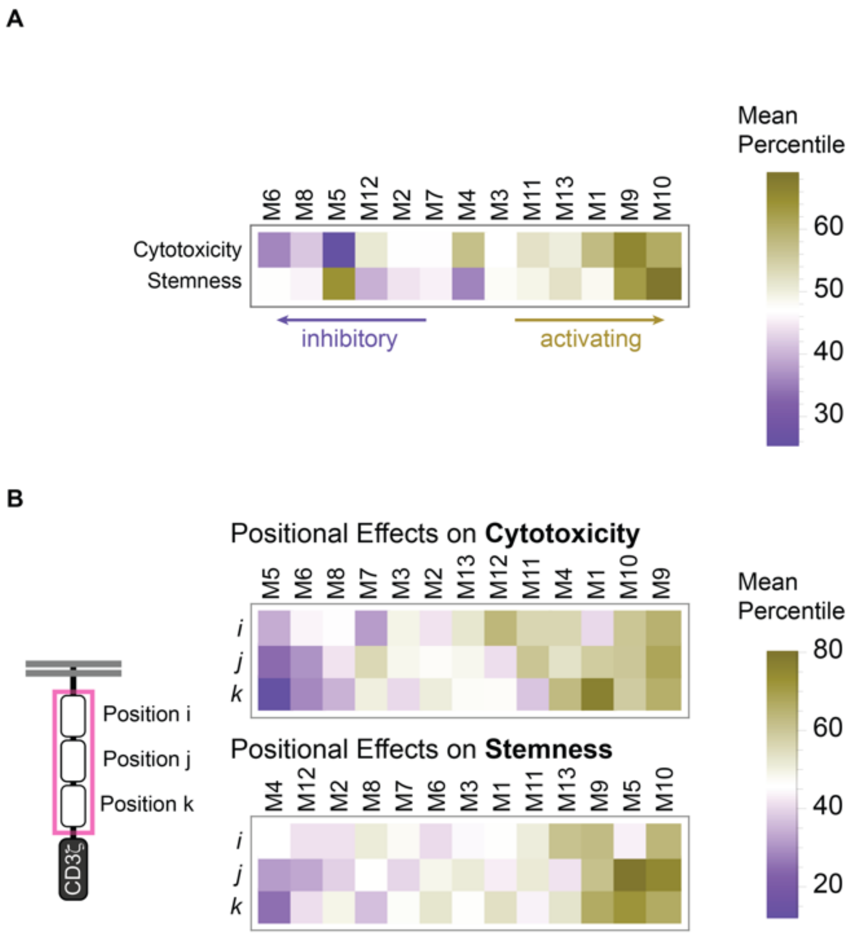

Figure S4. Distribution analysis quantifies elements of linear motif language to extract design parameters for signaling domains. **A,** Heatmaps of mean ranked percentile quantify the overall effects of library parts on CAR T cell cytotoxicity and stemness. **B,** Heatmaps of mean ranked percentile quantify the position-dependent effects of library parts on CAR T cell cytotoxicity and stemness.

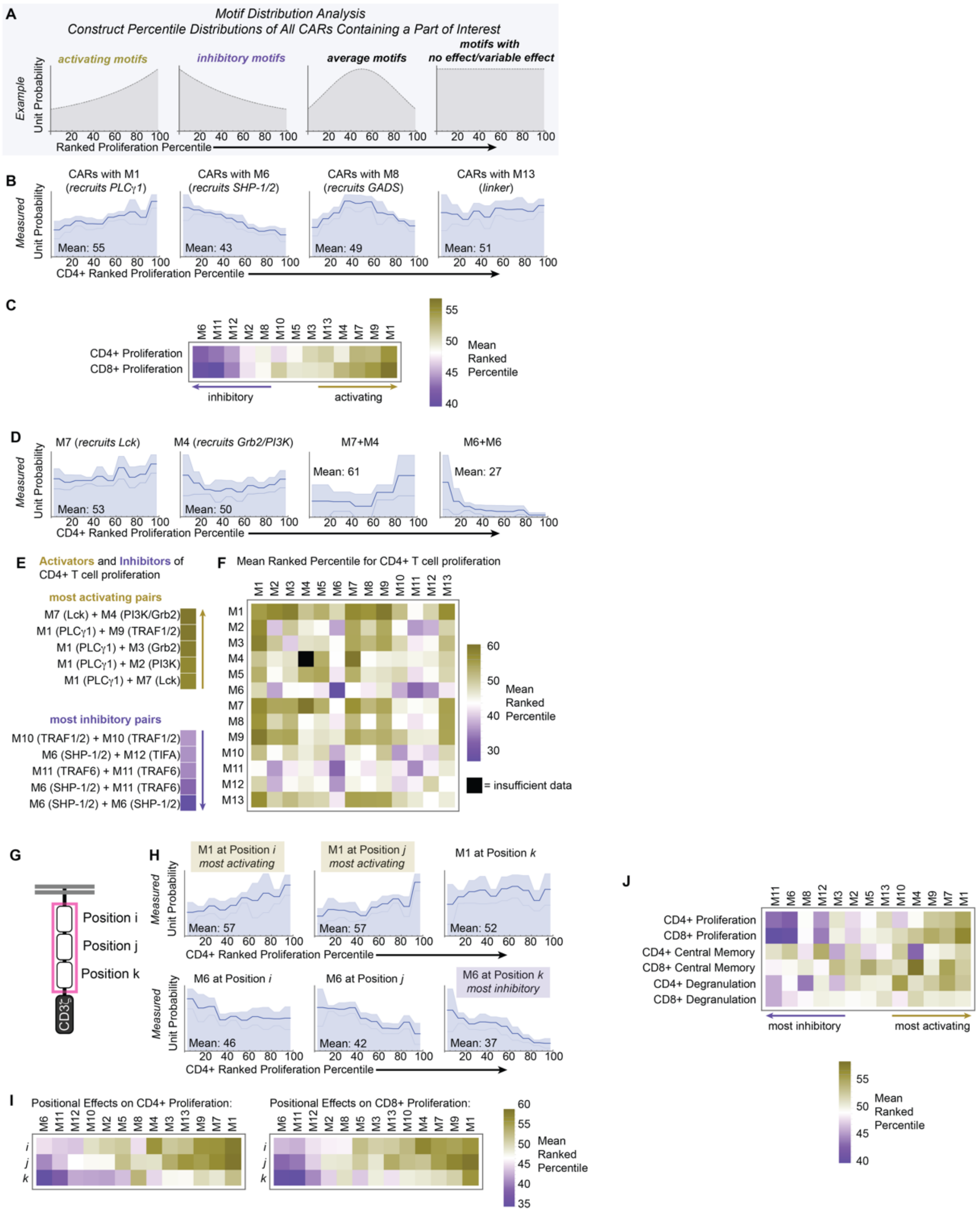

Figure S5. Distribution analysis quantifies contributions of library parts to CAR T cell proliferation in a pooled screen. A, Example percentile distributions for CARs that contain parts with various effects on CAR T cell phenotype. **B**, Percentile distributions from pooled screening demonstrate the varied effects of library parts on CD4+ T cell proliferation. The three lines within the distributions represent mean ± s.e.m. for n=3 pooled library screens. **C**, Heatmap of mean ranked percentile quantifies the overall effects of library parts on CAR T cell proliferation measured in pooled screens. **D**,

Example percentile distributions for CARs that contain individual parts or pairs parts. **E**, The most activating and most inhibitory pairs calculated using the means of percentile distributions. **F,** Mean ranked percentile for all pairs in the library. **G**, CAR schematic depicting positions *i*, *j*, and *k* in variable costimulatory domain. **H**, Percentile distributions from pooled screening demonstrate the position-dependent effects of M1 and M12 on CD4+ T cell proliferation. **I**, Heatmaps of mean ranked percentile quantify the position-dependent effects of library parts on CAR T cell proliferation measured in pooled screens. **J,** Heatmap of mean ranked percentile quantifies the overall effects of library parts on CAR T cell proliferation, central memory formation, and degranulation (a proxy for cytotoxicity) measured in pooled screens.

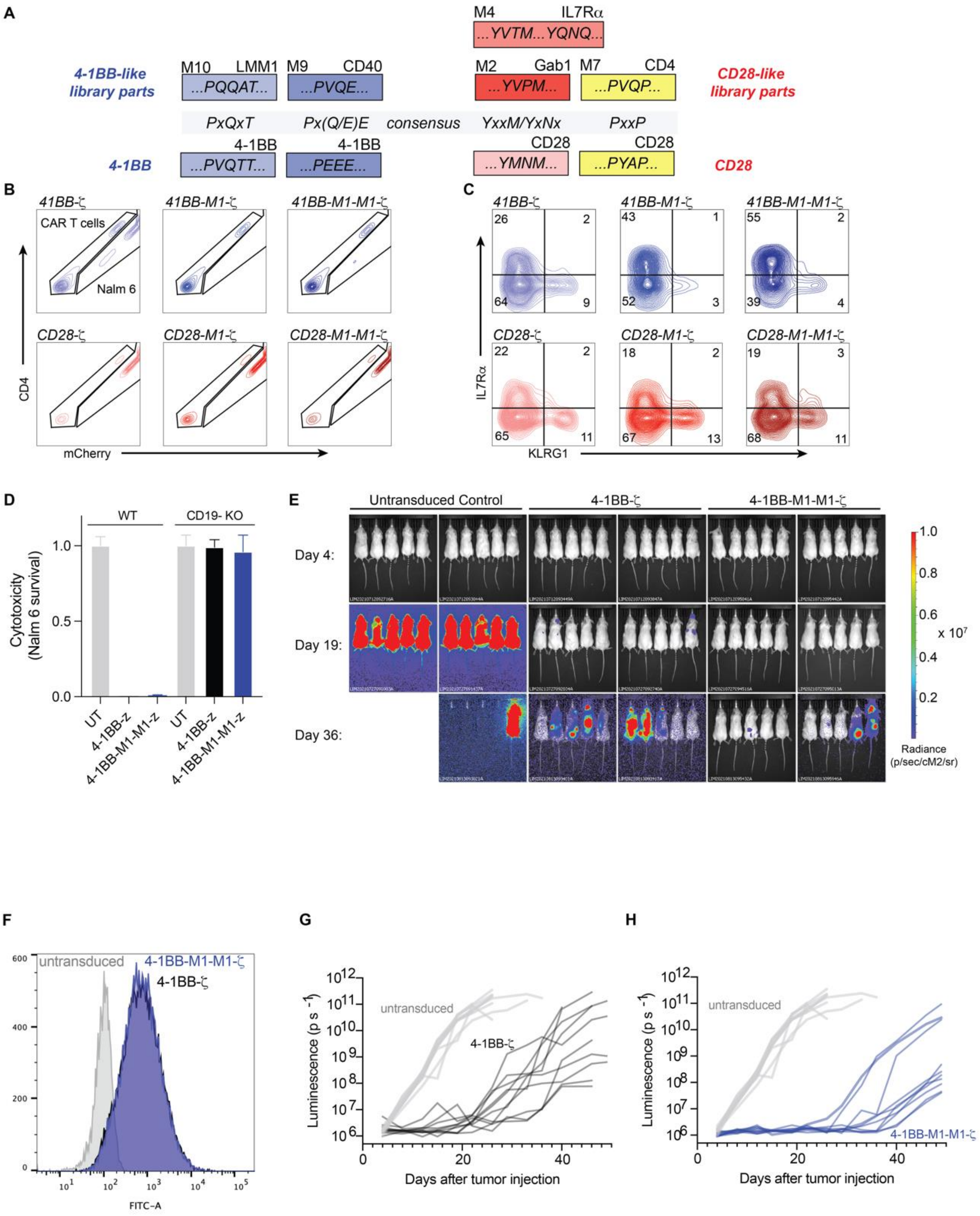

Figure S6. Neural networks accurately predict that PLCγ1 binding motifs improve the cytotoxicity and stemness of 4-1BB-ζ but not CD28-ζ. A, Schematics of signaling motifs in 4-1BB, CD28, and functionally similar library parts. **B-C**, *In vitro* assessment of the effect of adding one or two copies of M1 to 4-1BB and CD28 costimulatory domains. Cytotoxicity (B) and IL7Rα and KLRG1 expression (C) were assessed on day 9 after 4

challenges with Nalm 6 target cells. **D**, T cells with and without CAR were pulsed three times with wildtype Nalm 6 and CD19-negative Nalm 6 to assess off-target killing. T cells were cultured for 48 hours after final Nalm 6 pulse to maximize killing. **E,** Tumor progression was monitored using bioluminescent imaging of Nalm 6 expressing the firefly luciferase (fLuc) transgene. Scales are normalized for all time points. **F**, Transduction efficiency of sorted T cells used in mouse experiments was assessed by observing the FITC channel via flow cytometry on the day of injection into mice. FITC measurement reflects GFP expression in T cells with and without CAR-P2A-GFP transgenes. **G-H**, Individual luminescence traces for mice in **E**.

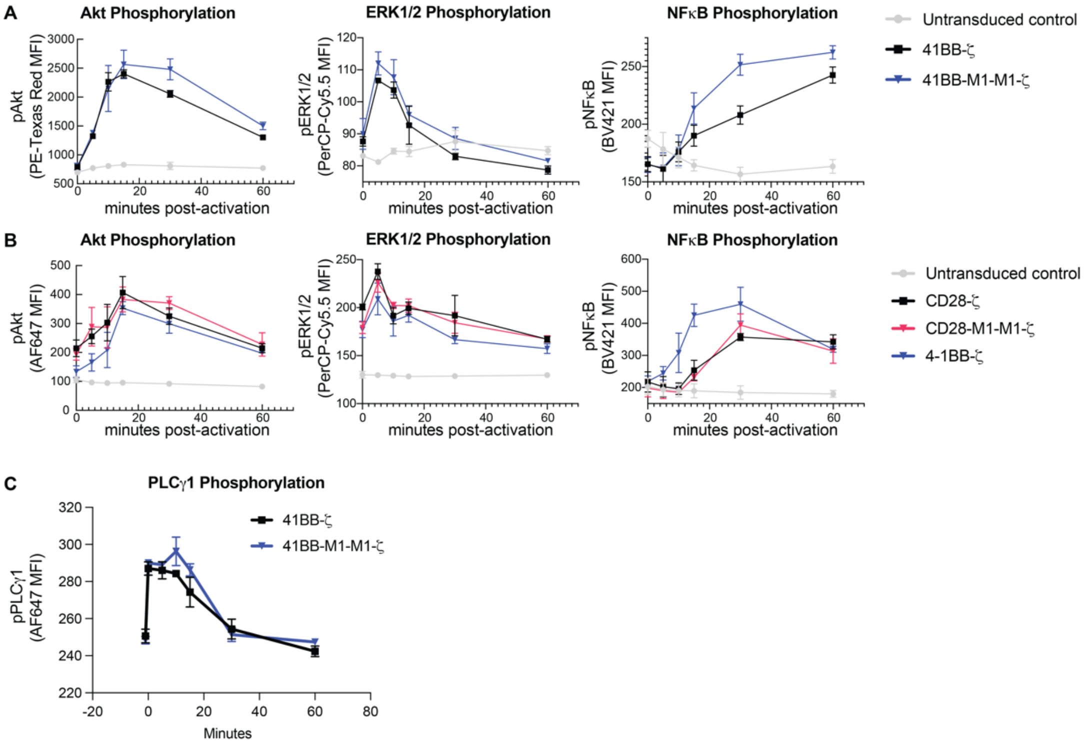

Figure S7. M1 increases Erk1/2 and NFκB phosphorylation in CAR T cells with 4-1BB-derived signaling domains. A-C, Kinetics of phosphorylation upon stimulation of CAR T cells with Nalm 6 target cells, measured by flow cytometry. Kinetic traces represent mean and standard deviation for n=3 replicates.

Neural Network Model	Convolutional Layer Filters	Convolutional Layer Kernel Size	LSTM Layer Units	Dropout Layer Dropout Rate	Fully Connected Layer Units
NN1:	20	5	4	0	64
NN2:	10	2	8	0	16
NN3:	20	4	8	0.2	4
NN4:	10	2	4	0	64
NN5:	20	4	2	0	16
NN6:	20	2	4	0	4
NN7:	10	5	4	0	16
NN8:	20	5	4	0	4
NN9:	20	2	4	0	4
NN10:	50	5	4	0	4

Table S1. Hyperparameters for neural networks trained on cytotoxicity data. Hyperparameters were selected by grid search as described in the methods.

Neural Network Model	Convolutional Layer Filters	Convolutional Layer Kernel Size	LSTM Layer Units	Dropout Layer Dropout Rate	Fully Connected Layer Units
NN1:	20	4	2	0	64
NN2:	20	4	2	0.1	4
NN3:	10	2	8	0	64
NN4:	10	4	2	0	64
NN5:	10	5	8	0	16
NN6:	10	3	8	0.1	64
NN7:	10	2	2	0	16
NN8:	50	4	4	0.2	4
NN9:	10	5	8	0	16
NN10:	20	2	4	0	4

Table S2. Hyperparameters for neural networks trained on stemness (%IL7Ra+/KLRG1-) data. Hyperparameters were selected by grid search as described in the methods.

Data S1. (DataS1ArrayAll.csv)

CAR library screening array data shown in Figures 1-3. Motifs are labeled 1 through 13 as shown in Figure 1C. Motif 17 corresponds to CD3z.

Data S2. (DataS2ArrayTraining.csv)

Training data set from CAR library screening array data shown in Figure 2. Motifs are labeled 1 through 13 as shown in Figure 1C. Motif 17 corresponds to CD3z.

Data S3. (DataS3ArrayTest.csv)

Test data set from CAR library screening array data shown in Figure 2. Motifs are labeled 1 through 13 as shown in Figure 1C. Motif 17 corresponds to CD3z.
